# Inter-population differences in salinity tolerance and osmoregulation of juvenile wild and hatchery-born Sacramento splittail

**DOI:** 10.1093/conphys/cov063

**Published:** 2016-02-16

**Authors:** Christine E Verhille, Theresa F Dabruzzi, Dennis E Cocherell, Brian Mahardja, Frederick Feyrer, Theodore C Foin, Melinda R Baerwald, Nann A Fangue

**Affiliations:** af1 Department of Wildlife, Fish, and Conservation Biology, University of California, Davis, CA 95616, USA; af2 Department of Animal Science, University of California, Davis, CA 95616, USA; af3 US Geological Survey, California Water Science Center, Sacramento, CA 95819-6129, USA; af4 Department of Plant Sciences, University of California, Davis, CA 95616, USA

**Keywords:** California, cyprinid, osmoregulation, salinity, splittail, wild

## Abstract

The Sacramento splittail (*Pogonichthys macrolepidotus*) is a minnow endemic to the highly modified San Francisco Estuary of California, USA and its associated rivers and tributaries. This species is composed of two genetically distinct populations, which, according to field observations and otolith strontium signatures, show largely allopatric distribution patterns as recently hatched juveniles. Juvenile Central Valley splittail are found primarily in the nearly fresh waters of the Sacramento and San Joaquin rivers and their tributaries, whereas San Pablo juveniles are found in the typically higher-salinity waters (i.e. up to 10‰) of the Napa and Petaluma Rivers. As the large salinity differences between young-of-year habitats may indicate population-specific differences in salinity tolerance, we hypothesized that juvenile San Pablo and Central Valley splittail populations differ in their response to salinity. In hatchery-born and wild-caught juvenile San Pablo splittail, we found upper salinity tolerances, where mortalities occurred within 336 h of exposure to 16‰ or higher, which was higher than the upper salinity tolerance of 14‰ for wild-caught juvenile Central Valley splittail. This, in conjunction with slower recovery of plasma osmolality, but not ion levels, muscle moisture or gill Na^+^,K^+^-ATPase activity, in Central Valley relative to San Pablo splittail during osmoregulatory disturbance provides some support for our hypothesis of inter-population variation in salinity tolerance and osmoregulation. The modestly improved salinity tolerance of San Pablo splittail is consistent with its use of higher-salinity habitats. Although confirmation of the putative adaptive difference through further studies is recommended, this may highlight the need for population-specific management considerations.

## Teaser

Genetically-distinct populations of the Sacramento Splittail, a minnow endemic to the San Francisco Estuary, exhibit different physiological responses and tolerances to salinity as juveniles. Population-level responses correspond with known salinity habitat differences suggesting some degree of local adaptation. Further studies are needed to elucidate these differences, but these data suggest a potential need for population-specific conservation and management strategies.

## Introduction

**Figure 1:**
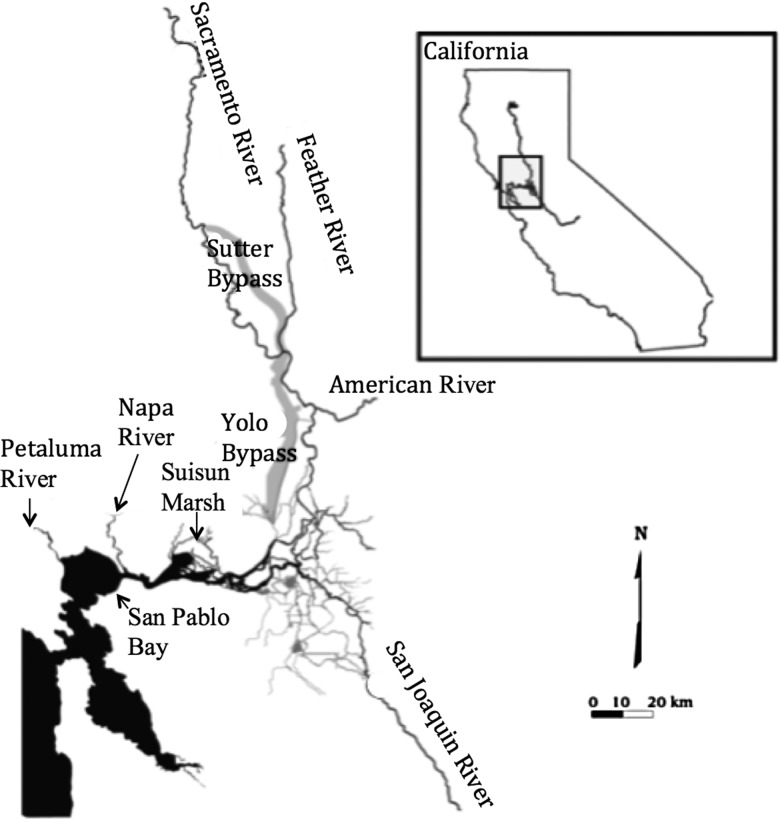
The San Francisco Estuary (in California) and its associated rivers and estuaries, with sampling locations and putative spawning locations for the wild San Pablo and Central Valley populations of Sacramento splittail (*Pogonichthys macrolepidotus*). The putative spawning locations for the San Pablo splittail population are the Napa and Petaluma Rivers. The putative spawning locations for the Central Valley splittail population are Suisun Marsh, the Sacramento and American Rivers and tributaries of the San Joaquin River. San Pablo splittail collections occurred in Napa and Petaluma Rivers. Central Valley splittail were collected at sites between the confluence of the American and Sacramento Rivers and Suisun Marsh.

The Sacramento splittail (*Pogonichthys macrolepidotus*) is a minnow endemic to the San Francisco Estuary and its associated rivers and tributaries in California, USA (Fig. 1). It is the only extant member of the *Pogonichthys* genus (Moyle, 2002; Moyle *et al*., 2004) and is composed of two genetically distinct populations referred to as the San Pablo and Central Valley populations (Baerwald *et al*., 2007, 2008). Although gaps remain in our knowledge of the geographical distribution, preferred habitat and physiology of splittail, preliminary findings suggest that San Pablo larvae often hatch in higher salinities than Central Valley larvae (Feyrer *et al*., 2010).

Sharp declines in the abundance of many San Francisco Estuary fish species over the last two decades have been linked to ecosystem-wide modifications (Moyle, 2002; Feyrer *et al*., 2003; Kimmerer, 2004; Sommer *et al*., 2007; Thomas *et al*., 2010). These modifications include the intrusion of salinity from the San Francisco Bay as a consequence of climate change and anthropogenic water consumption (Knowles and Cayan, 2002; Cayan *et al*., 2008; Cloern and Jassby, 2012). Evidence of declines in splittail population numbers (Meng and Moyle, 1995; Feyrer *et al*., 2003; United States Fish and Wildlife Service, 2003) resulted in federal listing of splittail as threatened under the US Endangered Species Act (United States Fish and Wildlife Service, 1999). Although the federal listing status was remanded in 2003, splittail retain classification as a species of Special Concern by the California Department of Fish and Wildlife.

Defining conservation and management plans for splittail is complicated by their complex semi-anadromous life history and the existence of two genetically distinct populations (Baerwald *et al*., 2007, 2008). Highly fecund spawning adults of both populations undergo an annual migration from the brackish, food-rich waters of the bays and marshes of the San Francisco Estuary to the spawning habitats (Feyrer and Baxter, 1998). Genotyping of age-0 splittail captured in the Napa and Petaluma Rivers (i.e. putative San Pablo population) and Suisun Marsh and the Sacramento–San Joaquin Delta (i.e. putative Central Valley population) suggests a primarily allopatric spawning distribution. Spawning and yearling rearing locations of the Central Valley splittail population (Fig. 1) are thought to be restricted to the freshwater tributaries and floodplains of the Central Valley draining the Sacramento and San Joaquin Rivers into the San Francisco Estuary, including San Pablo Bay (Daniels and Moyle, 1983; Sommer *et al*., 1997; Moyle *et al*., 2004; Baerwald *et al*., 2007, 2008). For the San Pablo population, spawning (Fig. 1) primarily occurs in the relatively brackish Napa and Petaluma Rivers (Baerwald *et al*., 2007, 2008). The hypothesized spawning distributions are supported by otolith strontium signatures of splitttail captured in San Pablo vs. Central Valley spawning locations, which show Central Valley splittail exclusively to inhabit water with salinities below 1‰ and San Pablo splittail to inhabit water of salinities up to 10‰ during the first 3 months of life (Feyrer *et al*., 2010). The differences in salinity between primary spawning/rearing habitats may suggest population-specific differences in salinity tolerance.

Fish in fresh water are hyperosmotic relative to the hypoosmotic water and approach isosmotic conditions at ∼10‰ (reviewed by Evans, 2008). As fish enter increasingly saline waters, they are faced with an osmoregulatory challenge where water is lost from their relatively hyposmotic blood across their gills into the hyperosmotic water (Evans, 2008). Water loss, which can be compensated for through drinking salt water, results in increased plasma osmolality and reduced muscle moisture; however, the ions gained must be excreted from the body to the external environment via the gills and kidneys. Ionocytes, called mitochondria-rich cells, are the main sites of sodium and chloride excretion in fish gills (Foskett and Scheffey, 1982; Perry, 1997; Evans *et al*., 2005). Na^+^,K^+^-ATPase (NKA) establishes the electrochemical gradients driving Na^+^ and Cl^−^ excretion at the mitochondria-rich cells and the kidneys of marine teleost fishes (Perry, 1997; Marshall, 2002). Other fish species, for example Atlantic killifish (*Fundulus heteroclitus*), distributed along an environmental salinity gradient display intraspecific plasticity in gene expression, plasma osmolality and ion responses to osmotic stress (Scott *et al*., 2004; Whitehead *et al*., 2011). Furthermore, salinity tolerance and physiological responses to osmotic stress vary ontogenetically in anadromous fishes (McCormick, 1994). Thus, fish exposed to increasingly saline waters experience ionic and osmotic disturbances, for which the capacity to compensate is limited and varies among life stages and populations.

Although putative responses of Central Valley splittail to osmotic disturbances have been studied (Young and Cech, 1996), they have never been compared with San Pablo splittail responses. To test the hypothesis that San Pablo and Central Valley splittail populations differ in their response to salinity during their juvenile life stage, we investigated salinity tolerance and the associated physiological responses of wild-caught and hatchery-born juvenile splittail exposed to variable salinities. We conducted a series of experiments including the following: (i) investigation of the effects of salinity on larval splittail growth; (ii) assessment of critical salinity tolerances of larval to juvenile splittail at exposure times ranging from hours to weeks; and (iii) an assessment of the osmoregulatory parameters (i.e. plasma osmolality, muscle moisture and NKA) describing the physiological responses of juvenile splittail to variable salinity exposures over time. The degree to which the two splittail populations exhibit different physiological responses to salinity may reflect local adaptation, the expression of which would be highly relevant to the establishment of effective conservation and management strategies for the species.

## Materials and methods

### Experimental fish

#### Capture of wild fish

Two age classes of splittail were collected at sites dispersed throughout the habitat range (Fig. 1). Young-of-year (after 50 dph) splittail were captured in beach seines or otter trawls from spring to summer of 2012 and 2013. When these fish exceeded 1 year of age, they are referred to here as ‘juvenile wild splittail’. Adult splittail, referred to here as ‘adult wild splittail’, were caught by gill netting or beach seining throughout the entire year in 2012, and spawned at the University of California, Davis (UCD) to produce hatchery-born splittail for salinity exposure experiments. All wild fish collections fell under California Department of Fish and Wildlife Scientific Collecting Permit SC-11901. All wild-captured splittail were acclimated to fresh water at UCD for at least 30 days before salinity exposures or spawning. Wild-captured fish were also genotyped to confirm their population of origin. See online supplementary material for details on wild capture, transport to UCD, acclimation to UCD laboratory conditions and genotyping. All fish handling, rearing and salinity tests were performed in agreement with the UCD Institutional Animal Care and Use Committee protocol no. 16788.

#### Larval/young-of-year hatchery-born offspring of wild-caught splittail

Adult wild splittail, caught in 2012, were genotyped to confirm their population of origin and spawned at UCD following the protocols of Deng *et al*. (2012). Spawning attempts were made with both San Pablo and Central Valley wild adults, but egg fertilization was only successful for the San Pablo population. The offspring of these hatchery spawns are referred to as larval (0–49 dph), YOY (50–360 dph) or juvenile hatchery-born San Pablo splittail, depending on the age at the time of experiments.

The fertilized eggs were incubated in tanks supplied with aerated, flow-through well water at 18.5 ± 0.5°C, with dead eggs being removed several times each day to prevent fungal infections. Larvae were fed a continuous supply of live *Artemia salinia* from 6 dph (i.e. at completion of yolk sac absorption) until 50 dph, when they were transitioned to a mix of HBH Baby Bites (HBH Pet Products Inc. Springville, UT, USA) and *Artemia salinia*. At 60 dph, fish were transitioned onto a mixture of HBH Baby Bites and Rangen Salmon Starter Diet, also supplied continuously throughout the day.

### Effects of salinity on splittail growth

The effects of salinity on growth of larval/YOY hatchery-born splittail of the San Pablo population were assessed from 13 to 69 dph. At 12 dph, 200 splittail were randomly allocated between four (50 fish per tank) 50 l static, aerated treatment tanks equipped with a sponge filter and maintained at 18.5 ± 0.5°C. After a 24 h adjustment period in the treatment tanks, water of two of the four tanks was gradually raised to 12‰ over 6 h at a rate of 2‰ h^−1^ by dissolving aquarium salt (Instant Ocean, Blacksburg, VA, USA) into the tank water. The 6 h salinity increase was consistent among all salinity exposures performed here, except for chronic lethal maximum and critical salinity maximum determinations. This duration of salinity increase was chosen to mimic a tidal cycle of San Francisco Bay, where the transition from low to high tide tends to occur in ∼6 h. Salinity was monitored in all experiments using a calibrated light refractometer and hand-held YSI 556 MPS (YSI, Yellow Springs, OH, USA). After the 6 h increase, salinity was maintained at 12‰ for 56 days (until fish reached the age of 69 dph), while the remaining two tanks were maintained at a salinity of 0.4‰ (fresh water). In order to maintain water quality, 30% of the water volume of both tanks was replaced daily with water of the appropriate salinity. Every 2 weeks, six fish were removed from each tank and terminally sampled for measurements of mass (in grams) and standard length (in millimetres).

### Splittail salinity tolerance experiments

#### Chronic lethal maximum of salinity

The chronic lethal maximum of salinity was assessed for YOY hatchery-born San Pablo splittail according to the protocol of Young and Cech (1996). At 59 dph, 150 juvenile splittail of mean mass 0.156 ± 0.009 g were randomly allocated between three 150 l tanks (one control and two salinity test tanks) and allowed to adjust to the new tanks for 24 h. The control tank was supplied with flow-through, aerated well water throughout the duration of the salinity test. The salinity test tanks were connected with a recirculation system to allow for manipulation of salinity, and both systems were maintained at 18–19°C. Beginning at 60 dph, the water salinity of the test tanks was gradually increased from acclimation salinity (0.4‰), at a rate of 0.08‰ h^−1^, by dissolving aquarium salt into the recirculation system. The test end point for each individual fish was loss of equilibrium, which was identified as when the fish was no longer able to maintain an upright position in the water. After loss of equilibrium, the fish was removed from the tank and measured for mass and standard length.

#### Critical salinity maximum

Critical salinity maximum was measured for larval (40 dph, *n* = 24) and YOY (80 dph, *n* = 24) hatchery-born San Pablo splittail according to the protocol of Young and Cech (1996). The mass of larval hatchery-born splittail was 0.074 ± 0.005 g and the mass of YOY hatchery-born splittail was 0.306 ± 0.018 g. Tests were performed in 7 l plastic test chambers partly submerged in a water bath with temperature controlled at 18.5°C. Individual fish were randomly assigned to an aerated test chamber 30 min before the beginning of the test. After the 30 min adjustment period, water salinity was increased with dissolved aquarium salt by 3–4‰ h^−1^ until fish lost equilibrium (Young and Cech, 1996). After loss of equilibrium, fish were immediately transferred to a recovery chamber with oxygenated, flow-through fresh water (18.5°C, 0.4‰). All fish recovered within 1 h. Approximately 24 h post-test, fish were removed from recovery chambers for measurements of length and mass.

#### Young-of-year and juvenile range-finding tests

**Figure 2:**
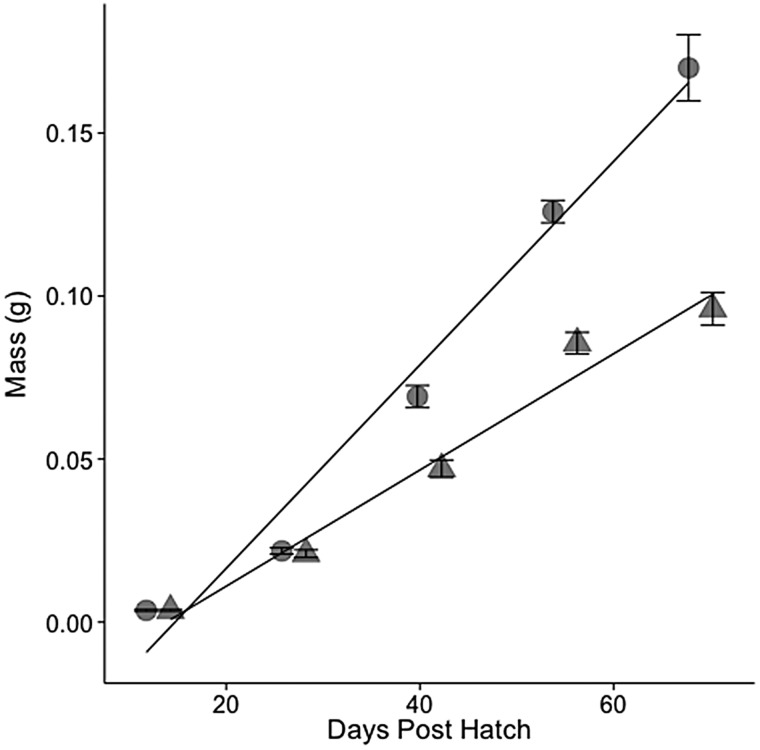
Growth of hatchery-reared larval to juvenile San Pablo population Sacramento splittail (*Pogonichthys macrolepidotus*) in fresh water (0.4‰; circles) and 12‰ (triangles) salinity water. Points are means ± SEM. Regression equations for fresh water and 12‰ salinity were *y* = −0.0498 + 0.0031*x* and *y* = −0.0224 + 0.0018*x*, respectively, where *y* is mass and *x* is days post-hatch. Points have been jittered in order to compensate for over-plotting. Sample size is two tanks (six fish per tank) for each time point.

Salinity range-finding exposures were performed to assess the upper value tolerated by pre-adult wild and hatchery-born splittail and to guide the salinities chosen for testing in the ‘*Experiments concerning the physiological response to salinity*’, detailed below. Fish used for these tests were 5.2 ± 0.4 g YOY hatchery-born San Pablo splittail, 9.6 ± 0.6 g juvenile wild Central Valley splittail and 14.8 ± 0.6 g juvenile wild San Pablo splittail. Salinities tested ranged from 11 to 20‰ (Table 1) for 2 weeks. The highest salinity tested in the range-finding tests (20‰) was based on the chronic lethal maximum of salinity of YOY hatchery-born San Pablo splittail (18–20‰; Table 2) and larval splittail growth impairment at salinities of 12‰ relative to fresh water (Fig. 2). Also taken into consideration were environmental salinity measurements at seine and trawl locations when capturing the wild fish used in the experiments reported here and published observations during wild capture suggesting that splittail are not found at salinities >16‰ in the wild (Feyrer *et al*., 2015).

**Table 1:** Summary of salinity range-finding tests for wild and hatchery-born Sacramento splittail (*Pogonichthys macrolepidotus*)

Salinity (‰)	Time (h)
Young-of-year San Pablo hatchery-born splittail	
16	>336
18	48
20	24
Juvenile wild Central Valley splittail	
11	>336
14	>336
16	168
Juvenile wild San Pablo splittail	
11	>336
14	>336
16	>336

Values in the ‘Time’ column indicate the number of hours for which survival, assessed as the ability to maintain an upright position in the water, remained at 100%.

**Table 2:** Summary of the chronic lethal maximum for salinity test for larval San Pablo hatchery-born Sacramento splittail (*P. macrolepidotus*)

Salinity (‰)	Time (h)	Survival (%)
Larval San Pablo splittail chronic lethal maximum for salinity		
0.4–18.0	0–216	100
20.0	240	89 ± 11
22.0	264	35 ± 27
24.0	288	1 ± 1
25.0	300	0

Salinity was increased from fresh water at a rate of 0.08‰ h^−1^ until all fish lost equilibrium. Salinity, test duration (in hours) and daily survival (expressed as a percentage ± SEM) are reported for every 24 h after the first loss of equilibrium was observed.

Range-finding tests were performed in 100 l recirculating tanks. Three to 20 test fish were moved from stock tanks into test tanks 24 h before testing to allow adjustment to test tanks. Testing began with a 6 h consistent salinity increase to the test salinity, using aquarium salt. Once the recirculation system water reached the test salinity, fish were monitored for loss of equilibrium for 336 h or until one fish within a tank lost equilibrium.

Once one fish lost equilibrium, a new tank of fish was tested at the next lowest salinity (Table 1). For example, YOY hatchery-born San Pablo splittail were first tested at 20‰. After the first fish lost equilibrium at 20‰, a new group of fish was exposed to 18‰ and monitored for loss of equilibrium. Once a fish at 18‰ lost equilibrium, 16‰ was tested. All fish within the 16‰ tank survived for 14 days at this salinity; therefore, lower salinities were not tested for YOY hatchery-born San Pablo splittail. Two fish that either showed opercular damage owing to contact with the tank cover or unusual scale loss over 50% of its body were excluded from this analysis.

### Experiments concerning the physiological response to salinity

#### Effects of salinity on osmoregulation of San Pablo hatchery-born splittail

The effects of salinity treatments on physiological variables reflecting osmotic balance at the whole-animal, cellular and tissue level of YOY hatchery-born San Pablo splittail were tested. Splittail were exposed to three salinity treatments (0.4, 12 and 16‰) for up to 336 h, with sampling time points of 0, 6, 12, 24, 72, 168 and 336 h (online supplemental material Fig. S1). The average mass of all fish sampled during this experiment was 254 ± 9 mg, and significant growth did not occur during the 2 week experiment.

Salinity treatments occurred in three independent recirculation systems, each dedicated to a different salinity level (0.4, 12 or 16‰). One week before salinity treatments began, fish were randomly allocated among 12 tanks (42 fish per tank, six tanks per population). During this adjustment period, all tanks were supplied with flow-through well water at 18.5 ± 0.5°C. Feed rations were maintained as in rearing tanks.

Salinity treatments began with increasing the salinity at a constant rate over 6 h until the treatment salinity of 12 or 16‰ was reached. All tanks, including the freshwater (0.4‰) control tank, which did not require a salinity adjustment, were transitioned from flow-through to recirculation water supply at the beginning of treatments to allow for salinity control. Recirculation systems were maintained at the treatment salinities for 336 h, with daily checks of salinity, dissolved O_2_, temperature and nitrogenous waste levels, including ammonia (salicylate test), total nitrite (polyethylene glycol) and total nitrate (polyethylene glycol sulfanilamide) concentrations (API Fresh and Saltwater Master Test Kits, Mars Inc., McLean, VA, USA).

At each sampling time point, six fish were randomly netted out of each tank (12 fish from each population at each salinity exposure time point) and anaesthetized in 0.5 g l^−1^ tricaine methanesulfonate (Argent Inc., Redwood, WA, USA) dissolved in well water and buffered with 2.5 g l^−1^ sodium bicarbonate. Once fish lost equilibrium, they were rapidly sampled for mass, fork length, blood, gill tissue and skeletal muscle. Samples were collected from all fish per tank within 10 min after introduction to anaesthetic.

Blood samples were collected by severing the caudal peduncle and drawing mixed blood directly from the caudal vein and artery into a heparinized microhaematocrit capillary tube (Precalibrated Micro-Hematocrit Tubes with Heparin; VWR, USA). Capillary tubes of blood were immediately centrifuged at room temperature, 20 000 *g* for 5 min, for haematocrit determination and plasma separation. Dorsal epaxial muscle samples weighing ∼100 mg were collected and placed on pre-weighed weigh boats for analysis of muscle water content.

##### Muscle moisture. 

The wet mass of skeletal muscle samples on pre-weighed weigh boats was measured within 30 min of dissection on a four-digit analytical balance, after which samples were desiccated for 7 days at 55°C. After desiccation, samples were reweighed, and muscle moisture was calculated as the percentage of wet mass lost with desiccation.

##### Haematocrit and plasma osmolality. 

Haematocrit (as a percentage) was measured immediately after blood collection on duplicate centrifuged microhaematocrit capillary tubes whenever possible, although a small number of fish were too small to collect sufficient blood to fill two tubes. Immediately after reading haematocrit, tubes were broken at the interface between blood and plasma to collect plasma into microcentrifuge tubes. Tubes were immediately flash frozen in liquid nitrogen and stored at −80°C until determination of osmolality. Plasma osmolality was measured on plasma samples equilibrated to room temperature using a Vapro™ Vapor Pressure Osmometer (model 5600) equipped with a mini sample holder to allow for small (2 µl) sample volumes. Whenever sufficient plasma was collected, osmolality measurements were performed in duplicate.

#### Effects of salinity on osmoregulation of wild San Pablo and Central Valley splittail

Population differences in the osmoregulatory response of juvenile wild splittail to salinity treatments were compared in San Pablo and Central Valley splittail at 11 and 14‰ salinity for up to 168 h (24, 72 and 168 h; Fig. S1). Given that mortalities occurred by 168 h when juvenile wild Central Valley splittail were held at 16‰ (Table 1), the highest salinity treatment level tested was 14‰. Salinity treatments were also compared with freshwater controls of both populations. Sample sizes of nine fish per population–salinity–time combination (not including freshwater control fish) were tested across a 30 day period from 10 April until 9 May, with control groups of five fish per population sampled on day 1.

Experimental protocols were similar to the ‘*Effects of salinity on osmoregulation of San Pablo hatchery-born splittail*’ described above, but were carried out in 20 l buckets plumbed to provide flow-through fresh water or recirculation system water. Two different buckets per population–salinity–time combination held four or five fish (total sample size of nine fish) randomly netted out of the stock tanks. The two salinities (11 and 14‰) were tested in series, with the 11‰ first, followed by 14‰. Salinity treatment durations were randomly allocated to adjacent pairs of buckets (one bucket per population) within the salinity recirculation systems. Fish were held in the experimental buckets supplied with recirculation system or flow-through fresh water (0.4‰) for a minimum of 3 days before salinity increases or sampling (for control fish). As in previous experiments, salinity treatments began with a linear 6 h increase in salinity to the treatment salinity, after which salinity was maintained within ±0.1‰ for the duration of the experiment. Feeding and water quality maintenance were as described in the ‘*Effects of salinity on osmoregulation of San Pablo hatchery-born splittail*’ described above.

At the end of the treatment period, water flow to the buckets was stopped, all but the bottom 5 cm of water (∼4 l) was removed, and 500 ml of a 11 g l^−1^ tricaine methanesulfonate solution buffered with 1 g l^−1^ sodium bicarbonate in water from the treatment tank was added to the bucket to an end concentration of 1 g l^−1^ of tricaine methanesulfonate and 0.1 g l^−1^ of sodium bicarbonate. Once fish lost equilibrium, they were rapidly sampled for mass, fork length, blood, gill tissue and skeletal muscle. Blood samples were collected from all fish immediately by a pair of researchers. Subsequent tissue sampling was identical to that of the pilot experiment on the physiological response to salinity, except that fin clips were collected and preserved in 100% ethanol for genotyping and that the larger size of fish in this experiment allowed for blood collection via caudal puncture. Additionally, for determination of NKA activity, the entire left side of the gill basket was collected into a 2 ml microcentrifuge tube filled with SEI buffer (250 mM sucrose, 10 mM Na_2_EDTA and 50 mM imidiazole, pH 7.3), flash frozen and then stored at −80°C until determination of NKA activity (∼6 months). Control fish were sampled in an identical manner to the salinity test fish.

#### Blood parameters. 

Blood samples were collected by caudal puncture using heparinized (1000 USP ml^−1^ heparin; APP Pharmaceuticals LLC, Schaumburg, IL, USA) 23-gauge hypodermic needles attached to 1 ml syringes (BD Precision Glide 23 G hypodermic needles; BD, Franklin Lakes, NJ, USA). The blood was injected into heparinized capillary tubes, which were processed as described in the ‘*Effects of salinity on osmoregulation of San Pablo hatchery-born splittail*’ experiment.

#### Na^+^,K^+^-ATPase activity. 

Gill NKA activity was quantified according to the methods of McCormick (1993) by spectrophotometrically measuring the rate of NADH loss through enzymatically coupling NKA-catalysed ATP hydrolysis with the oxidation of NADH to NAD catalysed by lactate dehydrogenase. Gill samples were thawed on ice and SEI buffer was replaced with 0.5–1 ml of fresh chilled 0.5% SEID buffer (20 ml SEI buffer, 0.1 g Na deoxycholic acid, pH 7.3), depending on the size of the gill tissue sample. Each sample was homogenized (Polytron; Kinematica AG, Lucerne, Switzerland) for 30 s, then immediately centrifuged for 30 s (4°C, 5000***g***; Eppendorf, Hamburg, Germany). The supernatant was immediately pipetted into 0.5 ml microcentrifuge tubes and held on ice for the brief period before enzyme activity readings. Ten minute spectrophotometric kinetic reads at 340 nm absorbance (25°C; Synergy HT microplate reader; Biotek, Winooski, VT, USA) were performed on triplicate 10 µl volumes of samples loaded into microplates, then combined with 200 µl of ouabain-positive or ouabain-negative assay solutions warmed to 25°C. Assay solutions were prepared and checked daily by determination of the ADP standard curve slope as described by McCormick (1993).

In order to standardize NKA enzyme activity to total protein among samples, total protein was quantified using a commercial test kit (BCA protein assay kit; Thermo Scientific, Rockford, IL, USA) according to the bicinchoninic acid technique (Smith *et al*., 1985). Finally, NKA activity (in micromoles of ADP per milligram of protein per hour) was calculated as the ouabain-inhibited fraction of total ATP hydrolysis and conversion of NADH to NAD^+^.

### Statistical analyses

Effects of salinity on larval growth were assessed using linear regression analysis. The lm function in R (R Core Team, 2014) was used to test the effects of dph, salinity and the dph*salinity interaction on mass. The values of mean critical salinity maximum of larval and YOY hatchery-born San Pablo splittail were compared using a *t*-test with the t.test function in R. Effects of salinity on osmoregulation of San Pablo hatchery-born splittail were tested for with separate three-way ANOVAs testing for main effects of salinity and time on plasma osmolality and muscle moisture. When significant main effects were seen, multiple comparisons were performed using Tukey’s *post hoc* test. Both tests were performed in R using the aov and TukeyHSD functions, respectively. When testing for effects of salinity on osmoregulation of wild San Pablo and Central Valley splittail, heterogeneity of variance within osmolality, muscle moisture and NKA activity among treatments was observed. Therefore, the effects of population, salinity and time at salinity were tested with a Kruskal–Wallis ANOVA using the kruskal function of the agricolae package of R (de Mendiburu, 2014). In order to reduce the number of factors from three to one for the Kruskal–Wallis ANOVA, population, salinity and time at salinity were all combined into one categorical ‘treatment’ factor with 14 levels {two populations × [(three time points × two salinities) + one time point for freshwater control salinity]}. Throughout this manuscript, data are reported as means ± SEM. For all statistical analyses, significance was determined based on an α of 0.05.

## Results

### Effects of salinity on splittail growth

The linear regression comparing larval splittail mass included a significant (*P* = 0.003) interaction between salinity and dph. The slope of the regression, and therefore growth rate, was 1.8-fold greater for larvae grown in 0.4 compared with 12‰ water (Fig. 2).

### Splittail salinity tolerance experiments

For the chronic lethal maximal salinity tests, no larvae lost equilibrium as salinity was increased from 0.4 to 18.0‰ over the first 219 h of the trial (Table 2). Approximately 10% of the fish lost equilibrium when salinity reached 20‰ at the 240th hour, and loss of equilibrium continued in both treatment tanks, with all fish losing equilibrium by the end of the 300th hour, when salinity reached 25‰. The critical salinity maximum for hatchery-born San Pablo splittail was higher than the chronic lethal maximal salinity and significantly decreased between 40 dph larval (32.2 ± 0.4‰) to 80 dph juvenile (29.4 ± 0.5‰) fish (*P* < 0.001). During range-finding tests, all juvenile splittail tolerated 14‰ water for at least 336 h without losing equilibrium (Table 1). Juvenile hatchery-born and wild San Pablo splittail were able to tolerate 16‰ for at least 336 h, whereas wild Central Valley splittail lost equilibrium within 168 h in 16‰ water.

### Physiological response to salinity ’experiments

**Figure 3:**
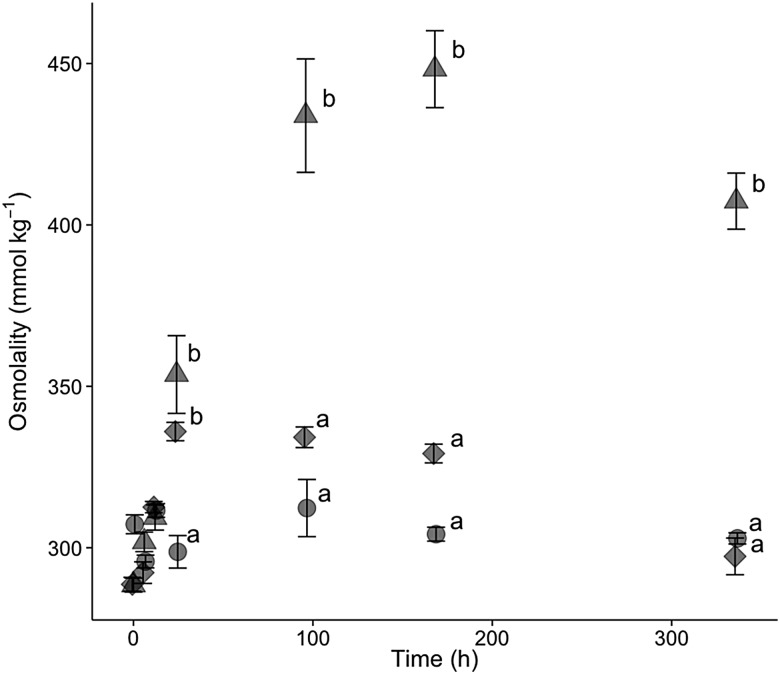
Plasma osmolality (means ± SEM) of hatchery-reared San Pablo population Sacramento splittail (*P. macrolepidotus*) exposed to fresh water (circles) and 12‰ (diamonds) and 16‰ (triangles) salinity water. Sample sizes ranged from eight to 18 fish per time point. Different letters demarcate significant differences between salinity treatments at the same time point.

**Figure 4:**
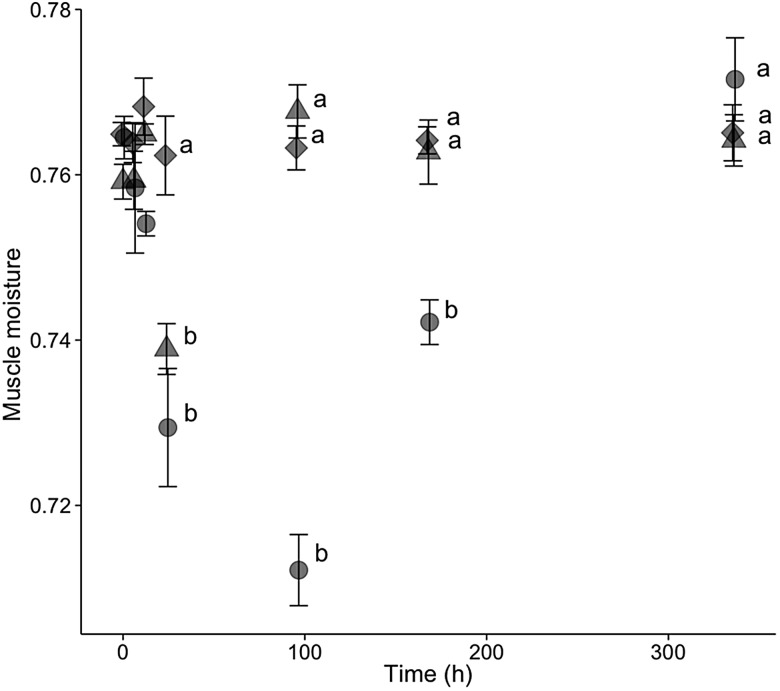
Muscle moisture (means ± SEM) of hatchery-reared San Pablo population Sacramento splittail (*P. macrolepidotus*) exposed to fresh water (circles) and 12‰ (diamonds) and 16‰ (triangles) salinity water. Sample sizes ranged from eight to 18 fish per time point. Different letters demarcate significant differences between salinity treatments at the same time point.

**Figure 5:**
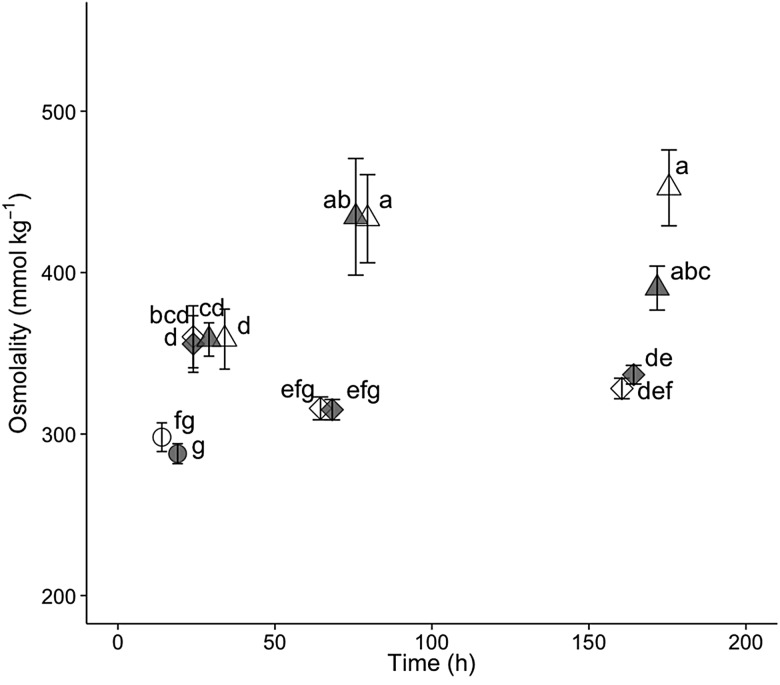
Plasma osmolality (mean ± SEM) of juvenile wild San Pablo (grey symbols) and Central Valley (open symbols) population Sacramento splittail (*P. macrolepidotus*) exposed to fresh water (circles) and 11‰ (diamonds) and 14‰ (triangles) salinity water. Sample sizes ranged from six to 11 fish per salinity treatment time point, and were five for control treatments. Different letters demarcate significant differences among all data points in the plot. Points have been jittered in order to compensate for over-plotting.

**Figure 6:**
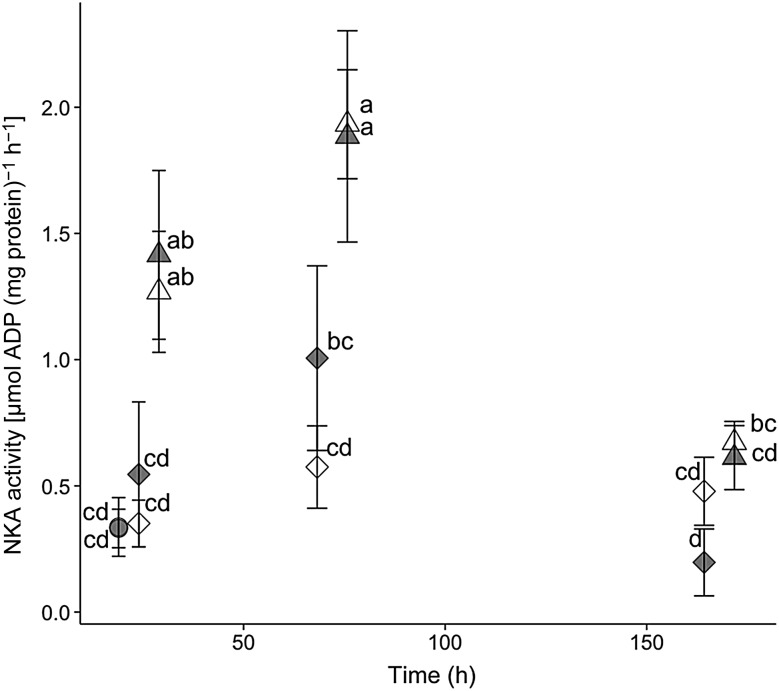
Gill Na^+^,K^+^-ATPase (NKA) activity (mean ± SEM) of juvenile wild San Pablo (grey symbols) and Central Valley (open symbols) population Sacramento splittail (*P. macrolepidotus*) exposed to fresh water (circles) and 11‰ (diamonds) and 14‰ (triangles) salinity water. Sample sizes ranged from six to 11 fish per salinity treatment time point, and were five for control treatments. Different letters demarcate significant differences among all data points in the plot.

**Figure 7:**
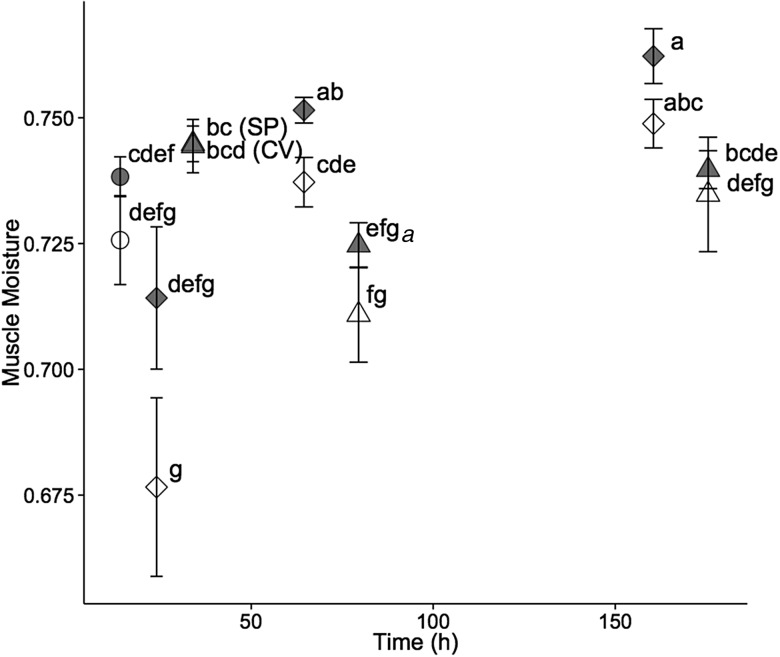
Muscle moisture (mean ± SEM) of juvenile wild San Pablo (grey symbols) and Central Valley (open symbols) population Sacramento splittail (*P. macrolepidotus*) exposed to fresh water (circles) and 11‰ (diamonds) and 14‰ (triangles) salinity water. Sample sizes ranged from six to 11 fish per salinity treatment time point, and were five for control treatments. Different letters demarcate significant differences among all data points in the plot. Abbreviations: CV, Central Valley splittail; and SP, San Pablo splittail.

**Figure 8:**
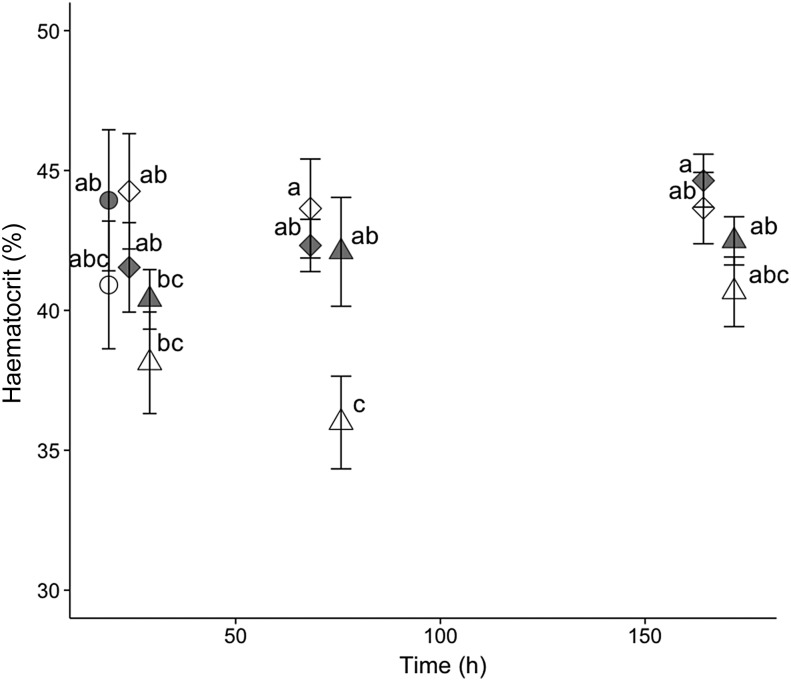
Haematocrit (mean ± SEM) of juvenile wild San Pablo (grey symbols) and Central Valley (open symbols) population Sacramento splittail (*P. macrolepidotus*) exposed to fresh water (circles) and 11‰ (diamonds) and 14‰ (triangles) salinity water. Sample sizes ranged from six to 11 fish per salinity treatment time point, and were five for control treatments. Different letters demarcate significant differences among all data points in the plot.

For hatchery-born San Pablo and wild San Pablo and Central Valley splittail, plasma osmolality, haematocrit, NKA activity and muscle moisture responses to salinity treatment followed the expected pattern of an initial disruption within 24–72 h, followed by a partial to full return to control values by 336 h of salinity treatment. The magnitude and duration of disruption to these osmoregulatory variables tended to be greater for the higher salinity level (Figs 3–8).

#### San Pablo hatchery-born splittail physiology

Plasma osmolality and muscle moisture of YOY hatchery-born splittail were significantly affected by salinity, time and an interaction between salinity and time (*P* < 0.001 for all six effects; Figs 3 and 4). The mean control value for osmolality was 307 ± 3 mmol kg^−1^ at time 0, and osmolality and muscle moisture did not significantly change over time. For both plasma osmolality and muscle moisture, significant differences between salinity treatments and controls were not observed until 24 h of salinity exposure. For the 12‰ treatment, plasma osmolality peaked at 336 ± 3 mmol kg^−1^ and muscle moisture reached its lowest value at 24 h. Osmolality and muscle moisture returned to control values by 96 h of the 12‰ salinity treatment. For the 16‰ treatment, the plasma osmolality peak of 448 ± 12 mmol kg^−1^ was at 168 h and muscle moisture was at its lowest value at 96 h. Muscle moisture returned to control values between 168 and 336 h, whereas osmolality remained elevated at 407 ± 9 mmol kg^−1^ at 336 h of 16‰ salinity treatment.

#### Wild San Pablo and Central Valley splittail physiology

In juvenile wild splittail, plasma osmolality increased significantly above control values within 24 h for both 11 and 14‰ treatments in Central Valley and San Pablo populations (Fig. 5). The 11‰ treatment returned to control values within 72 h for both populations, although a slight increase in plasma osmolality at 168 h resulted in a significantly greater value for the San Pablo but not the Central Valley population at that time point. For the 14‰ treatment, osmolality increased significantly above control values at 24 h and again at 72 h, with values at 168 h not differing significantly from those at 72 h for both populations. Osmolality of the San Pablo population at 14‰ decreased in magnitude between 72 and 168 h. Although this decrease was not significant, the 168 h osmolality was similar in magnitude, and significantly indistinguishable from the 24 h value, suggesting a fall in osmolality between 72 and 168 h for this population at 14‰.

Gill NKA activity generally reflected plasma osmolality and haematocrit, but with some notable differences (Fig. 6). For the 11‰ treatment, there was a small, insignificant and transient increase in NKA activity, which returned to control values by 168 h. Although the trend appeared more pronounced for San Pablo relative to Central Valley fish, the two populations did not differ significantly at any time point. For the 14‰ treatment, NKA activity was significantly elevated above control levels within 24 h and remained elevated until some time between 72 and 168 h for both populations.

The trends in muscle moisture response to salinity from 72 to 168 h, but not 24 h, generally reflected those of plasma osmolality, haematocrit and gill NKA activity (Fig. 7). For the 11‰ salinity exposure, there was a considerable but insignificant decrease in muscle moisture of both populations at 24 h, which is contradictory to the elevated plasma osmolality levels seen in both populations at 24 h. By 72 h, muscle moisture had increased to control levels for the Central Valley population, whereas muscle moisture of the San Pablo fish increased slightly to a value significantly greater than the controls. Both populations remained at similar levels of muscle moisture between 72 and 168 h and never differed significantly from each other during the entire trial at 11‰. Unlike plasma osmolality and gill NKA, muscle moisture for the 14‰ treatments never differed significantly from controls for either population. The general trend of a reduction in magnitude of muscle moisture at 72 h with a return to control levels by 168 h at 14‰ was consistent with the trends in NKA and osmolality.

Although haematocrit never varied significantly from controls at any time point for either population, the trends in response to salinity treatments reflected NKA and plasma osmolality patterns (Fig. 8). Haematocrit values at 11‰ remained similar in magnitude to control values for both populations from 24 to 168 h. For 14‰ salinity exposure, haematocrit of the San Pablo fish decreased in magnitude at 24 h, but returned to control values by 72 h. Haematocrit of the Central Valley fish decreased to a similar extent at 24 h, but with a further decrease at 72 h, resulting in a significantly lower haematocrit relative to the San Pablo fish and 11‰ Central Valley fish at this time point. By 168 h, the haematocrit of the Central Valley fish had returned to control values that were not significantly distinguishable from San Pablo values at the same time point.

## Discussion

We report the first comparison of the physiological responses of juvenile Central Valley and San Pablo splittail to salinity exposures. Salinity tolerance experiments as well as osmoregulatory indices reflecting physiological responses to salinity challenges provide equivocal support of our hypothesis that the San Pablo splittail population is more salinity tolerant than the Central Valley population. This potential intraspecific variation in response to salinity is consistent with the variation in habitat salinity occupied by the two populations of this species.

As predicted, larval San Pablo splittail experienced impaired growth at 12‰, relative to fresh water. This impaired growth suggests that salinity intrusion into larval rearing sites could be detrimental to the long-term performance of San Pablo splittail. Adult splittail spawn in upstream freshwater habitats from early March to mid-May, with larvae appearing in the spawning habitat in May and June (Daniels and Moyle, 1983) when, historically, river flows remained high, preventing salt water from reaching the upstream spawning habitat. However, for San Pablo splittail in the Napa and Petaluma rivers, upstream water diversions are increasing salt water intrusion and its occurrence earlier in the year. During larval splittail collections in May and June of 2002 and 2003, salinity ranged from 6 to 13‰ at Petaluma River capture sites and from 0 to 5‰ at Napa River capture sites (Baerwald *et al*., 2007). During the same years in the Central Valley rivers, salinity never exceeded 0‰. Unfortunately, we were unable to perform the salinity growth experiment on larval Central Valley splittail to determine whether the differences in habitat salinity between these populations translated into corresponding differences in larval performance responses to salinity.

Salinity range-finding experiments revealed that salinity tolerance differed between juvenile wild Central Valley and San Pablo splittail. Both wild and hatchery-born splittail exposed to experimental salinities over 336 h showed a maximal tolerable salinity of 16‰ for San Pablo splittail and 14‰ for Central Valley splittail. In general, these salinity tolerance values are in the range previously reported for similar-sized juvenile wild splittail (1–4 g) captured in putative Central Valley population locations and assessed for time to loss of equilibrium at salinities ranging from 14 to 28‰ (Young and Cech, 1996). Young and Cech (1996) showed that the onset of loss of equilibrium at 14‰ for 1–4 g splittail did not occur within the maximal exposure duration of 120 h. This is comparable to the juvenile wild Central Valley splittail here, which did not lose equilibrium during 336 h at 14‰. Juvenile wild Central Valley splittail tested here at 16‰, which lost equilibrium within 168 h, were also comparable to previously reported 1–4 g splittail tested at the same salinity, which lost equilibrium within 50 h (Young and Cech, 1996). The 1–4 g splittail tested by Young and Cech (1996) were of a similar size to the YOY hatchery-born San Pablo splittail tested here, which did not lose equilibrium during the entire 336 h exposure period at 16‰. The salinity tolerance of the YOY hatchery-born and juvenile wild San Pablo splittail tested here was more similar to the larger splittail (19–42 g) tested by Young and Cech (1996), which did not lose equilibrium during the entire 120 h exposure to 16‰. Thus, salinity tolerances determined from salinity range-finding tests corresponded to previously reported salinity tolerances of Central Valley splittail and suggest comparatively higher salinity tolerance for San Pablo splittail.

Although the osmoregulatory response to salinity has never been assessed in splittail, the magnitudes of plasma osmolality, muscle moisture, NKA activity and haematocrit values for wild juvenile splittail at 14‰ were similar to those of many fish species, including white sturgeon (*Acipenser transmontanus*; McEnroe and Cech, 1985; Mojazi Amiri *et al*., 2009), Italian sturgeon (*Acipenser naccarii*; Cataldi *et al*., 1995; Martinez-Alvarez *et al*., 2005), Gulf of Mexico sturgeon (*Acipenser oxyrinchus*; Altinok *et al*., 1998) and shortnose sturgeon (*Acipenser bravirostrum*; Jarvis and Ballantyne, 2003; Ziegeweid and Black, 2008). Maximal values of plasma osmolality (452 ± 23 ’mmol kg^−1^) and NKA activity [1.9 ± 0.2 µmol ADP (mg protein)^−1^ h^−1^] for 14‰ treatments are comparable to those of channel catfish (*Ictalurus punctatus*), for which plasma osmolality and NKA activity increased to ∼450 mmol kg^−1^ and 3.5 µmol ADP (mg protein)^−1^ h^−1^, respectively when held in 40% sea water (∼13‰ salinity; Eckert *et al*., 2001).

The general trends in the osmoregulatory response to salinity treatments were consistent with those seen in other species and similar for both wild populations at both test salinities. With salinity treatment, physiological variables tended initially to depart (i.e. increase for plasma osmolality and NKA and decrease for muscle moisture and haematocrit) from control values, then gradually return to control or close to control values as the fish compensated for the osmoregulatory disturbance over time. The magnitude of departure from control values tended to be greater and the onset of return to control values tended to be later in fish at the higher (14 and 16‰) relative to lower (11 and 12‰) salinities. The exception of muscle moisture at 24 h salinity treatment is notable. At this time point, the muscle moisture of both populations of wild splittail dropped in magnitude at 11‰ but not at 14‰. However, this drop was not significant, and the expected greater drop for 16‰ relative to 12‰ occurred in the hatchery-born San Pablo splittail. Therefore, this exception may simply reflect random variation in muscle moisture.

Importantly, inter-population comparisons of the timing and magnitude of the physiological response to salinity treatments provides modest evidence of higher salinity tolerance for the San Pablo relative to the Central Valley splittail population. For the 11‰ salinity treatment, the physiological response was largely identical for San Pablo and Central Valley splittail for all osmoregulatory variables assessed. In contrast, for the 14‰ salinity treatment, the magnitude of osmoregulatory departure from control values tended to be greater for Central Valley compared with San Pablo fish. Muscle moisture was lowest for both populations at 72 h, reaching a lower value in Central Valley compared with San Pablo fish. Increases in osmolality and reductions in haematocrit and muscle moisture are suggestive of osmotic water loss at the whole-animal, cellular and tissue levels, respectively, although peak NKA activity and osmolality values at 24 and 72 h were similar for both populations. Despite the greater magnitude of changes in Central Valley osmoregulatory variables, differences between the two populations were not often statistically significant.

In contrast to the similar magnitudes of osmoregulatory disturbance in both splittail populations exposed to salinity treatments, the timing of recovery differed significantly between Central Valley and San Pablo splittail populations. The more rapid physiological recovery of homeostasis for San Pablo splittail suggests improved salinity tolerance for this population. San Pablo osmolality values at 168 h were statistically indistinguishable from the 24 h values of the same population, whereas Central Valley 168 h values were still significantly higher than their 24 h values. Likewise, haematocrit values for Central Valley splittail at 14‰ were still lower in magnitude than control values at 72 h, when San Pablo values had already begun to rise back to control values. Thus, our osmoregulatory assessments were consistent with salinity range-finding experimental results, which showed greater salinity tolerance for the San Pablo population over the Central Valley population.

A necessary step to connect conclusively the apparent differences in salinity tolerance between populations shown here to a genetic basis would be to conduct common garden experiments on fish from both populations. We were able to produce hatchery-born San Pablo splittail, but not Central Valley splittail, allowing us to hypothesize about, but not conclusively test, the role of environmental vs. genetic influences. If previous environmental exposures influenced the response of wild splittail to our laboratory salinity exposures, juvenile wild San Pablo splittail, which inhabit waters of higher salinity than the Central Valley population, would probably have had higher salinity tolerance than their freshwater hatchery-born counterparts. Here, the maximal tolerable salinity did not differ between hatchery-born and wild San Pablo splittail, suggesting that previous salinity exposures in the wild contributed little to the salinity responses described here. Therefore, it seems unlikely that any differences in the salinity tolerance and physiological responses to osmotic challenges observed between San Pablo and Central Valley splittail populations are attributable to environmental exposure. It follows that these differences in salinity tolerance may have a genetic basis, and heritability studies in conjunction with comparisons of the cellular mechanisms associated with differing salinity tolerances could confirm evidence of and determine the level of phenotypic variation caused by genetic variation.

Effective conservation and management of this fish species of concern requires understanding of phenotypic differences and gene flow between Central Valley and San Pablo splittail populations as well as their habitat requirements. Although the two populations are genetically distinct, large numbers of juvenile Central Valley splittail are sporadically captured in Napa and Petaluma Rivers (Baerwald *et al*., 2008; Mahardja *et al*., 2015), presenting the potential for substantial gene flow between the two populations. However, a decade of genetic sampling shows no significant change in the genetic distinctiveness of wild Central Valley and San Pablo splittail populations (Mahardja *et al*., 2015). If significant gene flow has been occurring between the two populations, the small San Pablo population would certainly show Central Valley genetic influences. Here, we showed some support for phenotypic differences in salinity tolerance that correspond to differences in salinity of the spawning habitats of the two populations. The match between salinity tolerances and spawning habitat salinities is congruous with evidence of little gene flow between Central Valley and San Pablo splittail populations. Central Valley and San Pablo splittail phenotypes, genotypes and habitat characteristics suggest that sporadic detections of juvenile Central Valley splittail in putative San Pablo spawning habitat are due to rapid dispersal of Central Valley splittail from their spawning habitat when salinity conditions through corridors connecting Central Valley and San Pablo habitats are favourable, and not a result of sympatric spawning.

Salinity tolerance and physiological responses to salinity of juvenile Central Valley and San Pablo population splittail were consistent with the prediction that San Pablo splittail are more salinity tolerant than Central Valley splittail, although the magnitude of the differences was modest. This study provides some evidence supporting the correspondence of variation in phenotype (i.e. salinity tolerance and physiological responses to salinity exposures) and habitat of the two populations, and suggests that the two populations may represent distinct evolutionarily significant units, with limited gene flow occurring between them. These findings highlight possible population-specific management considerations for these fish. Currently, the United States Fish and Wildlife Service has not listed the San Pablo distinct population segment (DPS) as threatened, and splittail is treated as a single species listed only as a species of special concern. The San Pablo DPS is genetically distinct, with a considerably smaller effective population size compared with Central Valley fish (Mahardja *et al*., 2015). The potential selective advantage the San Pablo DPS may have in increasingly saline conditions in the San Francisco Estuary points to it being a potential reservoir for survival of this species faced with increasing upstream salinity intrusion (Cloern and Jassby, 2012) in essential habitats of both population segments. The small effective population size and potential as a reservoir for the splittail species may warrant special consideration for protection of the San Pablo DPS.

## Supplementary material

Supplementary material is available at *Conservation Physiology* online.

## Funding

The research was funded by the Delta Science Program (grant number 2037 to N.A.F., M.R.B. and T.F.) and the University of California, Davis Agricultural Experiment Station (grant number 2098-H to N.A.F.).

## Supplementary Material

Supplementary Data
