# Supplementary material for: Inter-population differences in salinity tolerance and osmoregulation of juvenile wild and hatchery-born Sacramento splittail
Source: Conserv Physiol. 2016 Feb 16;4(1):cov063. doi: 10.1093/conphys/cov063 (PMC4758839; doi:10.1093/conphys/cov063)
Supplement: Supplementary Data [file cov063supp.zip › cov063supp.docx]

## Supplemental Section

Adult Wild Splittail Capture:

Wild adult splittail were captured by gill netting or beach seining. Beach seines were 10-m in length with a stretch mesh size of 6 mm. Gill nets (2 cm^2^ mesh size) set for two hr, and continuously monitored to ensure fish were removed within two min of entanglement.

YOY/Juvenile Wild Splittail Capture:

YOY wild splittail were captured by beach seine netting and/or otter trawling. The otter trawls were performed for 5 to 10 min, depending on water channel size, and at 4 km/hr using a four-seam trawl with a 1.5 m by 4.3 m opening and 5.3 m length. Mesh size was 35 mm stretch in the body and 6 mm stretch in the cod end. Repeated fishing of reputed San Pablo population spawning habitat was conducted in the Napa River within 2 km up or down stream of the city of Napa (Figure 1). The Petaluma River was not sampled because during all visits, salinity levels were 19‰, or higher, which is well above those expected to be tolerable for either population (Young and Cech, 1996). Sampling for splittail in the Napa River was conducted with beach seines from June 20 to July 18 in 2012 and July 17 to August 20 in 2013. As the reputed spawning habitat of the Central Valley population is more extensive than that of the San Pablo population, 24 sites throughout the Suisun Marsh and Sacramento and American Rivers were sampled from early June to mid-August of 2012 and 2013. In total, 21 sites in nine sloughs within Suisun Marsh and two sites at the confluence of the Sacramento and American Rivers (Discovery Park) were sampled (Figure 1).

After capture, fish were held in buckets of fresh water from the capture site containing Novaqua® (Kordon LLC, Hayward, CA, USA) until transport to the University of California, Davis (UCD). Just before leaving the water system to transport fish to UCD, fish were gathered into a sealed bag filled with fresh water adjusted to capture site salinity with Instant Ocean (Instant Ocean, Blacksburg, VA, USA) if necessary. Transport bags were filled with pure oxygen and conditioned with Novaqua®. Transport time to UCD never exceeded two hours, transport water temperature always remained between 19 to 22°C, and oxygen saturation of transport water never fell below 80%.

Upon arrival at UCD, bags were floated in one of two 150 l holding tanks for 30 min to one hr, during which the water in the transport bags was gradually diluted with tank water to equilibrate fish to tank temperature and salinity. The reputed San Pablo and Central Valley fish were held in separate tanks. Tanks were supplied with 18 to 19°C flow-through, degassed well water (conductivity 670 µS cm^-1^, dissolved O_2_ >6.0 mg l^-1^, and pH 8.1). For the first two weeks in captivity, a prophylactic regime of daily 1‰ salinity and oxytetracycline (as additive to feed) treatments were administered to prevent disease outbreaks. For the prophylactic salinity treatment, aquarium salt was dissolved into the tank water to raise the salinity to 1‰, while fresh water flow into the tank was maintained to allow a gradual dilution of salt to fresh water. Fish were held in captivity for a minimum of 30 days before use in any experimental manipulation.

Genotyping:

All wild-caught fish were genotyped to confirm population assignments. DNA was extracted from caudal fin tissue using the QIAGEN DNeasy 96 kit (QIAGEN Inc.) according to manufacturer’s protocols. A total of eighteen microsatellite markers were amplified for population genetic assignment: CypG3, CypG4, CypG23, CypG25, CypG35, CypG39, CypG40, CypG43, CypG45, CypG48, CypG52, CypG53, Pmac1, Pmac4, Pmac19, Pmac24, Pmac25, and Pmac35 (Baerwald and May, 2004; Mahardja *et al*., 2012). PCR and allele scoring procedures followed Mahardja *et al*. (2014). We used STRUCTURE 2.3.3 (Pritchard *et al*., 2000) to genetically assign individuals to their putative population. Previously genetically assigned YOY splittail described in Baerwald *et al*. (2007) and Mahardja *et al*. (2014) served as references for the two populations. STRUCTURE analysis was performed for ten iterations at K = 2 with all individuals and references included, no prior location information, 500,000 burn-in period, and 1,000,000 Markov chain Monte Carlo repetitions under the assumption of admixture and correlated allele frequencies. Replicate runs were averaged in CLUMPP 1.1.2 (Jakobsson and Rosenberg, 2007) with the FullSearch algorithm. Based on the efficiency and accuracy scores found in Vӓhӓ and Primmer (2006), we selected an average q-value of 0.8 as the threshold for distinguishing between purebred individuals and potential hybrids or unassigned individuals.


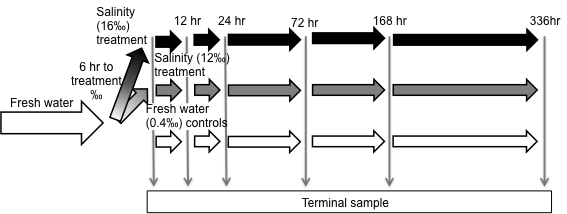


Figure S1. Experimental design for measuring the osmoregulatory disturbances caused by salinity exposure for Young of the Year hatchery-born San Pablo splittail (*Pogonichthys macrolepidotus*). Sample sizes were 12 fish per salinity-time-sample condition. The *Salinity Effects on Osmoregulation of Wild San Pablo and Central Valley Splittail* experiment followed a similar design, but test salinity levels were 14‰, 11‰, terminal sample time points were 24, 72 and 168 hours and sample sizes were 9 fish per population-salinity-time-sample condition. Controls (n=5) were sampled only at the 24 hr time point. See written methods for more details on sampling.
